# Einthoven dissertation prizes 2016

**DOI:** 10.1007/s12471-017-0978-z

**Published:** 2017-04-11

**Authors:** J. J. Piek

**Affiliations:** Department of Cardiology, Academic Medical Centre, Amsterdam, The Netherlands

The dissertation prize is named after Willem Einthoven who was a pioneer in cardiovascular medicine and recorded a human ECG for the first time in 1902, for which he was awarded with the Nobel prize in 1924 [1]. This yearly event is an initiative of the Netherlands Heart Institute (NHI) and the Netherlands Society of Cardiology (NVVC) to select the best three cardiovascular theses published in the year 2016. The jury received a total of 19 PhD dissertations for selection. The ranking of the theses was based upon a combination of parameters that included the curriculum vitae of the candidate, the scientific originality of the PhD thesis as well as its relevance for the cardiovascular field. Moreover, several objective bibliometric parameters were used that included the number of articles in citation index journals, both in PubMed and the Web of Science (WOS), the number of citations in WOS, the Hirsch index and finally the contribution of the candidate as a first author. Based upon this evaluation the jury elected the following nominees: Lourens Robbers, Romy Franken and Michiel de Graaf. The members of the jury were Prof. P.A. Doevendans and Prof. D.J. Duncker (Netherlands Heart Institute), Prof. B.J.M. Mulder (NVVC), Prof. J.W. Deckers (Director CVOI) and Prof. M.J. Schalij (President Concilium Cardiologicum). The three candidates presented their PhD theses at the annual spring congress of the NVVC held at De Leeuwenhorst in Noordwijkerhout on 6–7 April 2017. We congratulate the laureates for their excellent scientific work and their presentations during the meeting.

## Summary

### Defining microvascular injury in acute myocardial infarction and response after cell therapy using cardiovascular magnetic resonance imaging

Although mortality rates for acute myocardial infarction have decreased over the years, the number of patients suffering from heart failure after myocardial infarction is increasing. Early recognition of patients at risk for heart failure is necessary to optimise treatment. Cardiovascular magnetic resonance (CMR) imaging allows the accurate depiction of cardiac morphology, function, perfusion and tissue composition. Although CMR has already established itself as a valuable tool in clinical practice, new CMR techniques may provide additional information on the severity of the injury and expected recovery.

In the first part of this thesis, novel CMR techniques were addressed to further evaluate the composition of the myocardium after PCI-treated myocardial infarction. A few days after myocardial infarction, T1 and T2* values are increased in the infarcted myocardium. However, if microvascular injury (MVI) was present, T1 and T2* values significantly decreased in the infarcted area, showing that T1 and T2* values need to be interpreted carefully in the presence of MVI.

Additionally, we further investigated the tissue composition of areas with MVI by using a porcine model of reperfused myocardial infarction. Seven days after inducing a myocardial infarction by temporary coronary artery occlusion, CMR with T2-weighted imaging and late gadolinium enhancement imaging was performed, directly followed by histological examination of the myocardium. This showed that CMR-defined areas of MVI contain extensive necrosis with extravasation of erythrocytes, and complete destruction of the vasculature, while the surrounding contrast-enhanced area showed necrosis with intact microvessels containing microthrombi. The new insight into the relation of MVI, T2-defined haemorrhage and the histological findings shows that the phenomenon of MVI may not be caused by microvascular thrombotic ‘obstruction’ alone and allows further research into strategies aiming to preserve the microvasculature to attenuate damage.

Thirdly, CMR showed us that LGE-defined infarcted tissue heterogeneity can help in predicting the occurrence of ventricular tachycardia (VT) on 24-hour Holter monitoring 1 month after the infarction. Larger proportions of grey zone, or penumbra, were associated with an increased risk of VTs, supporting the theory that infarct heterogeneity may facilitate VT development. As risk stratification for effective prophylactic ICD implantation remains debated, further studies into the infarct composition may help in identifying the patients who might benefit from ICD therapy.

The second part of this thesis assessed the effects of intracoronary cell therapy after myocardial infarction on perfusion recovery and long-term functional recovery. In the first days after the infarction, perfusion differed between the infarcted myocardium and remote, unaffected myocardium. These regional differences persisted after 4 months and no difference in perfusion recovery was found for any treatment group in any of the regions. At long-term follow-up, no additional functional improvement was found from cell therapy on left ventricle function, suggesting that cell therapy does not provide benefit in its current form as an adjuvant treatment strategy after myocardial infarction.

In conclusion, the thesis shows the versatility of CMR and its necessity for daily cardiology practice. In the future, it may provide us with new tools to identify patients with more severe myocardial damage due to infarct-related haemorrhage, and patients with increased risk of developing arrhythmias after infarction. Further assessment of infarct tissue characteristics and the relation with functional recovery may provide more tailored therapy for patients after myocardial infarction.

*L.F.H.J. Robbers*

*VUMC*

*Email:* *lrobbers@spaarnegasthuis.nl*

## Summary

### Marfan syndrome: getting to the root of the problem

In this thesis we have tried to get to the root of the problem of the Marfan syndrome (MFS). We obtained more insight into the diagnosis, prognosis and markers of aortic disease, we explored novel treatment strategies to prevent aortic complications and obtained more knowledge about the importance of genetics in MFS. Currently, the diagnosis of MFS is established by the revised Ghent criteria, which includes the family history, an *FBN1* mutation, aortic dilation (Z-score ≥ 2), aortic dissection, ectopia lentis and a list of systemic features. However, we have shown that these criteria do not apply to populations other than Caucasian, resulting in underdiagnosis of MFS.

Aortic dissection impacts the lifespan of MFS patients negatively. Previously, we identified MFS patients at risk for type B aortic dissection using the aortic diameter, prior aortic root surgery, aortic distensibility and an aortic dilation rate above 0.5 mm/year. We have now added two novel predictors for aortic complications. An increase in the tortuosity index of the aorta as well as an elevated plasma TGF-β level lead to an increased aortic dilation rate and an increased risk for aortic dissection.

After the introduction of prophylactic aortic root replacements, the life expectancy has been increased tremendously. However, despite surgery, MFS patients still have an increased risk of premature death by aortic dissection. In order to reduce the aortic dilation rate and subsequently delay the need for prophylactic aortic root surgery, patients are pharmacologically treated with β‑blockers, which reduce aortic wall stress. However, β‑blockers do not completely prevent aortic complications, thus novel treatment strategies are still necessary. We have shown increased vascular inflammation in the aortic root of adult MFS mice, which is significantly reduced by treatment with losartan or anti-inflammatory drugs. However, besides losartan, anti-inflammatory agents did not reduce the aortic dilation rate in MFS mice.

In a prospective, randomised, controlled trial, we confirmed the beneficial effects of losartan treatment on aortic root dilation rate in adults with MFS. Furthermore, we could demonstrate a reduction in aortic dilation rate of the aortic arch in the subgroup of patients with a history of aortic root surgery. We found large variability in individual aortic root dilation rates upon losartan treatment. This and other highly variable phenotypic appearances are a result of the large number of different *FBN1 *mutations.

After reviewing the universally available *FBN1* mutation database, we are now able to classify mutation in the *FBN1 *gene based on their actions at the DNA level into less fibrillin-1 protein (haploinsufficiency (HI)) or less functioning (mutated) fibrillin-1 protein (dominant negative (DN)) incorporated in the aortic wall. We have found and confirmed that patients with an HI-*FBN1* mutation are at increased risk for aortic dissection and cardiovascular mortality, compared with patients with a DN-*FBN1* mutation. Furthermore, a difference in therapeutic response was shown. Losartan significantly reduces the aortic root dilation rate in MFS patients with an HI-*FBN1* mutation, whereas only a modest insignificant reduction is found in those with a DN-*FBN1* mutation, which highlights the importance of genetics in MFS.

*R. Franken*

*AMC*

*Email: r.franken@amc.nl*

## Summary

### Computed tomography coronary angiography: from quantification of coronary atherosclerosis to risk stratification of patients

In the past 10 years coronary computed tomography angiography (CTA) has emerged as a widely used non-invasive imaging test for the detection of coronary artery disease (CAD); its clinical applicability is widely incorporated in the current guidelines. Especially the high sensitivity (~98%) allows for accurate rule-out of disease in patients with suspected CAD. In the current literature, there is a growing body of evidence supporting the clinical and prognostic relevance of coronary plaque burden in addition to the presence of obstructive CAD. Using coronary CTA several features of coronary atherosclerosis can be identified; besides the evaluation of luminal narrowing, the extent, composition and location of CAD can be assessed. Using novel software algorithms these dimensional and compositional parameters can be (automatically) quantified. The aim of this thesis was to explore the value of coronary CTA in clinical practice, specifically targeting the feasibility of quantitative assessment of coronary atherosclerosis on CTA (QCT) and the clinical applicability of quantitative parameters of atherosclerosis.

First, the ability of QCT to quantify coronary plaque composition was assessed. QCT-derived parameters were compared with invasive measurement derived from intravascular ultrasound virtual histology. Both dimensional and compositional parameters were well correlated between the two modalities. Thus, automatic characterisation of coronary atherosclerosis with QCT is feasible and accurate.

Second, the value of QCT for risk stratification of patients was established. For this purpose all QCT-derived detailed data on the location, severity and composition of atherosclerosis were incorporated in a single score. After more than 2 years follow-up of patients with stable chest pain, the CTA risk score was significantly higher in patients who experienced a major adverse cardiac event (MACE). More importantly, all MACE occurred in patients with a high CTA risk score. It seems this CTA risk score allows accurate risk stratification of patients with suspected CAD.

A drawback of coronary CTA is the fact that the haemodynamic significance of a lesion cannot be evaluated. In this thesis it was demonstrated that quantitative parameters provided better correlation with the presence of myocardial ischaemia on myocardial perfusion imaging with SPECT as compared with current visual assessment of coronary CTA.

Using coronary CTA to assess changes over time in coronary atherosclerosis can be evaluated. For clinical practice, disease progression or regression is an important variable which could be used to evaluate the efficacy of medical therapy, but also provide more detailed insight into the natural history of coronary atherosclerosis. In the coming years, QCT will play in pivotal role in the quantification of disease progression.

Additionally, the clinical value of coronary CTA in the specific setting of high-risk diabetic patients without chest pain syndrome was established. With regards to the specific setting of high-risk diabetic patients without chest pain, several conclusions can be derived from this thesis. First, if treated with optimal medical therapy, very few patients present with progression of myocardial ischaemia. Second, the prognosis of these patients is good; the overall long-term event rate is limited. Especially diabetic patients without CAD on coronary CTA have an excellent prognosis. Even though the prognostic value of CTA was demonstrated in this thesis, it is unclear if screening using cardiac imaging influences the outcome of these patients. Additionally, cardiac CTA or CAC score could help tailor medical therapy in this challenging patient population.

*M.A. de Graaf*

*LUMC*

*Email:* *m.a.de_graaf@lumc.nl*

Michiel de Graaf won the first prize, Romy Franken the second prize and Lourens Robbers the third prize.
